# Rapid and selective concentration of bacteria, viruses, and proteins using alternating current signal superimposition on two coplanar electrodes

**DOI:** 10.1038/s41598-018-33329-7

**Published:** 2018-10-08

**Authors:** Chang-Ho Han, Seong Yong Woo, Jyoti Bhardwaj, Abhinav Sharma, Jaesung Jang

**Affiliations:** 1 School of Mechanical, Aerospace and Nuclear Engineering, Ulsan National Institute of Science and Technology (UNIST), Ulsan, 44919 Republic of Korea; 2 Department of Biomedical Engineering, UNIST, Ulsan, 44919 Republic of Korea; 3 School of Materials Science and Engineering, UNIST, Ulsan, 44919 Republic of Korea

## Abstract

Dielectrophoresis (DEP) is usually effective close to the electrode surface. Several techniques have been developed to overcome its drawbacks and to enhance dielectrophoretic particle capture. Here we present a simple technique of superimposing alternating current DEP (high-frequency signals) and electroosmosis (EO; low-frequency signals) between two coplanar electrodes (gap: 25 μm) using a lab-made voltage adder for rapid and selective concentration of bacteria, viruses, and proteins, where we controlled the voltages and frequencies of DEP and EO separately. This signal superimposition technique enhanced bacterial capture (*Escherichia coli* K-12 against 1-μm-diameter polystyrene beads) more selectively (>99%) and rapidly (~30 s) at lower DEP (5 Vpp) and EO (1.2 Vpp) potentials than those used in the conventional DEP capture studies. Nanometer-sized MS2 viruses and troponin I antibody proteins were also concentrated using the superimposed signals, and significantly more MS2 and cTnI-Ab were captured using the superimposed signals than the DEP (10 Vpp) or EO (2 Vpp) signals alone (*p* < 0.035) between the two coplanar electrodes and at a short exposure time (1 min). This technique has several advantages, such as simplicity and low cost of electrode fabrication, rapid and large collection without electrolysis.

## Introduction

Dielectrophoresis (DEP) refers to the movement of polarizable particles in a non-uniform electric field^1,2^. This technique is an effective means of manipulating a specific type of biological particle, for example, particular species^3^, size^4^, or life state^5^ in a heterogeneous particle mixture^6^. Coplanar electrodes such as interdigitated electrodes have been widely used to generate DEP due to their simple fabrication and ease of analysis. However, the DEP force over such electrodes generally decreases exponentially with the height above the electrode surface^7^; hence it is usually effective close to the electrode surface^8,9^. Furthermore, it is not easy to manipulate nanometer-sized biological particles, such as viruses and proteins, rapidly by using DEP because the dielectrophoretic mobility decreases with the square of the particle diameter^6^.

On the contrary, alternating current (AC) electroosmosis (EO) is fluid motion induced by electrode polarization when AC electric potentials are applied to planar microelectrodes at intermediate characteristic frequencies^10^. This technique has been applied in several fluidic applications, such as micropumps with arrays of asymmetric electrodes^11–13^ and micromixers for chemical species and electrolytes^14–16^ with low applied electric potentials. As AC EO is exerted on fluids rather than particles, the use of AC EO alone may be limited in several applications such as sorting, separation, selective concentration, and focusing based on their electrical properties.

In this regard, DEP and EO need to be combined to enable the selective and rapid concentration of particles far from the electrodes as well as near the electrodes onto a particular spot, such as a sensing element, thereby increasing the sensitivity of a sensor to biological particles such as bacteria, proteins, and viruses^17,18^. Few studies have been conducted using both EO and DEP on planar electrodes; in those studies, two electrodes generating DEP were implemented in the gaps between two outer electrodes inducing EO^19,20^. Therefore, the shapes and sizes of the planar electrodes were limited, and more care needs to be taken to avoid electrical shorts between the electrodes.

Other studies in which a pair of sinusoidal signals between two planar electrodes were used have also been reported on; in those cases, both DEP and EO needed to occur at the same AC frequency^17,21–24^ or to be generated in alternating time intervals^25^ (Table 1). However, AC EO generally occurs when the frequency is less than a few kHz^26^ while DEP capture usually works effectively in higher frequencies, for example, kHz or MHz regions^27,28^; therefore, applying a pair of sinusoidal signals to generate both DEP and EO may not work in many cases. Moreover, there are risks in this case that electrode damage or air bubble generation may occur by electrolysis at low frequencies^29^, when the electrical potential needs to be increased for better particle manipulation.

**Table 1:** Electrokinetic studies involving simultaneous treatment of DEP and EO, and signal superposition.

	Method	Particle	Medium	AC Excitation & Exposure Time	Electrode Type (Width/Gap)	Electrode Material	Reference
DEP + EO	Narrow DEP electrodes inside wide EO electrodes	1-μm-diam. PS beads	DI water	DEP: 1 Vpp, 1731 Hz, EO: 1 Vpp, 1000 Hz, 60 s	IDEs (EO: 30 μm/25 μm; DEP: 1 μm/1 μm)	Ti/Au	^19^
	Circular DEP-EP electrodes inside wider ring-shaped EO electrodes	*Staphylococcus aureus*	300 mM sucrose solution	DEP-EP: 0.5 V DC bias, EO: 8–12 Vpp, 800 Hz, 3 min	Planar rings	Au	^20^
	Two sinusoidal signals (0° & 180°)	*Escherichia coli*	1.5 M AHA (relative permittivity: ~200)	20 Vrms, 400 kHz, 12 s	IDEs (35 μm/35 μm)	Ti/Pt	^21^
	Two sinusoidal signals (0° & 180°)	*Saccharomyces cerevisiae*	0.1 mM NaHCO_3_ (9.15 μS/cm)	6.74 Vrms, 1 kHz, 10 min	Top–bottom	ITO	^22^
	Two sinusoidal signals (0° & 180°)	CTB	10 μM phosphate buffer	1 Vpp, 47 Hz, 20 min	Coplanar (7 μm gap)	Ti/Au/Ti	^17^
	Four sinusoidal signals (0°, 90°, 180° & 270°) for travelling-wave	HL-60 cells	100 μM KCl + 5% glucose (18 μS/cm)	3 kHz, 12 min	Circular sectors	Ti	^23^
	Two sinusoidal signals (0° & 180°)	25-μm-diam. barium titanate particles	2-propanol (0.011 μS/cm)	15 Vpp, 60 Hz, 8 s	IDEs (60 μm/40 μm)	Ti	^24^
	Alternately applied DEP and EO signals (DEP- > EO- > DEP- > EO- > …)	1-μm-diam. latex particles	DI water (0.25 μS/cm)	DEP: 1.6 Vpp, 5 kHz, EO: 1.6 Vpp, 300 Hz, 5 min	Square spiral (5–30 μm/5 μm)	Cr/Au	^25^
Signal Superposition for DEP	DEP + twDEP (sine + sine superposition)	T lymphocyte	Sucrose solution (400 μS/cm)	DEP: 5.2 Vpp, 30 kHz, twDEP: 5.6 Vpp, 350 kHz, unknown time	IDEs (10 μm/10 μm)	—	^30^
	DEP + DEP (sine + sine superposition)	Viable/non-viable yeast cells	Diluted PBS (600 μS/cm)	DEP (focusing): 3.39 Vrms, 60&90 kHz, DEP (sorting): 4.38 Vrms, 5 MHz, 20 s	Liquid electrodes chambers (20 μm/20 μm)	Ti/Pt	^31^
	DEP + electrorotaion (sine + sine superposition)	T lymphocyte	Inositol-added medium (326 μS/cm)	DEP (trap): 2 Vpp, 20 kHz, electrorotation: 0.4 Vpp, 100 kHz, 30 s	3D octode (top–bottom quadrupoles; 50 μm gap)	Cr/Au	^32^
	Pulsed DEP (sine + on-off cycles)	3-μm-diam. PS beads	DI water	Sine: 20 Vpp, 10 MHz, on-off: 0.3 Hz, 10 s	IDEs (30 μm/30 μm)	ITO	^33^
	Pulsed DEP (sine + on-off cycles)	Single lambda-DNA	DI water (1.1 μS/cm)	Sine: 20 Vpp, 1 MHz, on-off: 20 Hz, 1–10 s	Coplanar (10 μm gap)	Silicon nanotweezers	^34^
	Pulsed DEP (sine + square superposition)	10-μm-diam. PS beads	DI water (2 μS/cm)	Sine: 10 Vpp, 50 kHz, square: 10 Vpp, 2 MHz, 1 s	Top–bottom	ITO	^35^
DEP + EO via Signal Superposition	DEP + EO (sine + sine superposition)	*E. coli* K-12/1-μm-diam. PS beads	0.01× PBS (184 μS/cm)	DEP (selective concentration): 5 Vpp, 2 MHz, EO (convection): 1.2 Vpp, 1633 Hz, 30 s	Coplanar (25 μm gap)	ITO	The present study
		MS2 virus	Diluted in DI water (4 μS/cm)	DEP (concentration): 10 Vpp, 100 kHz, EO (convection): 2 Vpp, 1000 Hz, 1 min			
		Troponin I antibody	Diluted in DI water (16 μS/cm)	DEP (concentration): 10 Vpp, 10 kHz, EO (convection): 2 Vpp, 500 Hz, 1 min			

PS: polystyrene; EO: electroosmosis; DEP: dielectrophoresis; IDEs: Interdigitated electrodes; EP: electrophoresis; AHA: azidohomoalanine; ITO: indium tin oxide; CTB: cholera toxin subunit B; twDEP: traveling-wave DEP; PBS: phosphate buffered saline; DI: deionized.

Here, we present a simple and effective method of concentrating bacteria, viruses, and proteins rapidly and selectively on two coplanar electrodes via superimposing AC DEP and EO signals for biosensor applications. Signal superposition techniques have been previously employed in several studies using two sets of sinusoidal waves^30–32^, and pulsed sinusoidal waves^33–35^; however, they involved only electrical forces exerted on the particles suspended in fluids such as DEP, traveling wave DEP, and electrorotation for sorting, separation, trapping etc., and hence many of the particles located far from the electrode could not be manipulated rapidly. In the present study, we combine EO with DEP to enhance particle capture selectively and rapidly, which is critical for biosensors requiring rapid detection of biological particles. Although EO and DEP have been extensively studied (Table 1), this topic has not attracted much attention^36^.

**Figure 1:**
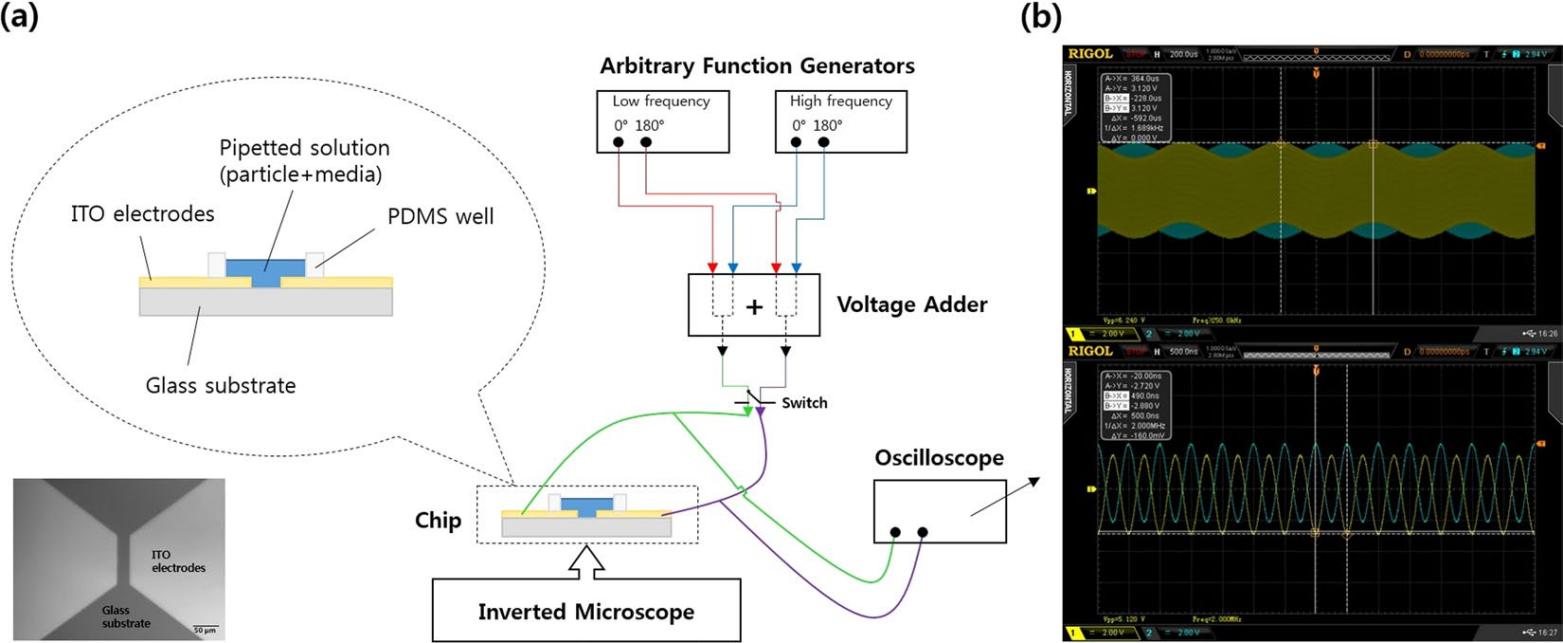
(**a**) Schematic of the experimental setup. (**b**) Superimposed signals: 1.2 Vpp (peak-to-peak), 1633 Hz signal for EO, and 5 Vpp, 2 MHz signal for DEP. Lower frequency (top) and higher frequency (bottom) components of the superimposed signals are shown^36^.

Firstly, the frequencies for optimal EO and DEP generation were determined, and the two waveforms were superimposed and applied to the fabricated coplanar electrodes (Fig. 1). Selective concentration of *Escherichia coli* K-12 was conducted against 1-μm-diameter polystyrene (PS) beads from a bacteria–bead mixture. MS2 viruses and fluorescein isothiocyanate (FITC)-labelled troponin I antibody (cTnI-Ab) were also tested to determine whether this method would work for nanometer-sized particles. The particles concentrated within targeted areas on the electrodes were quantified with different treatments: no solution (negative control), no signal (positive control), lower frequency signals (EO), higher frequency signals (DEP), and superimposed signals (EO + DEP), and the effects of these treatments were analyzed.

## Results and Discussion

**Figure 2:**
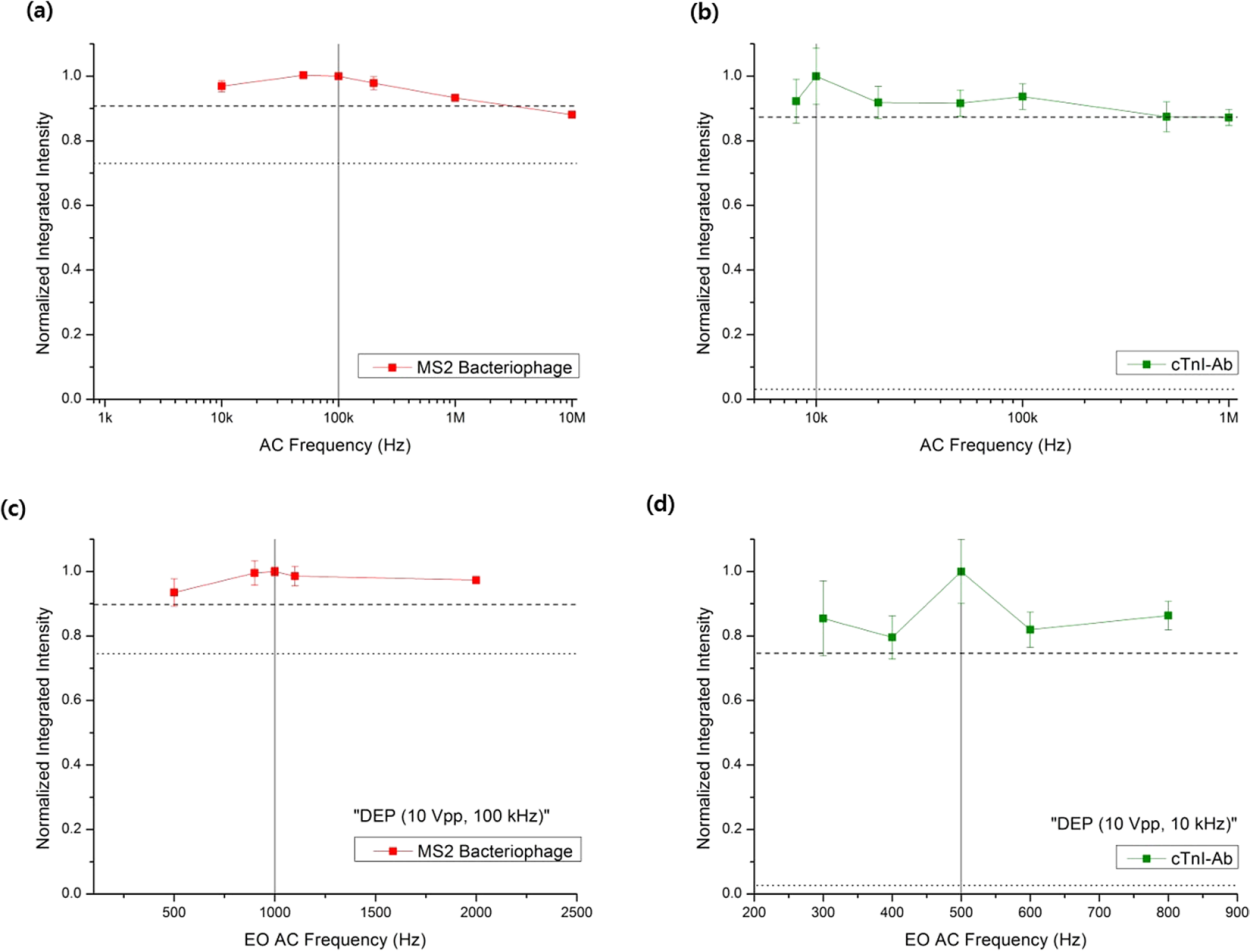
Experimentally investigated DEP capture behavior with an applied electrical potential of 10 Vpp for (**a**) MS2 viruses in DI water (4 μS/cm) and (**b**) cTnI-Ab in DI water (16 μS/cm), where the dashed and dotted horizontal lines indicate positive and negative control values, respectively. Experimentally investigated capture behaviors of (**c**) MS2 viruses, and (**d**) cTnI-Ab, when varying the EO frequencies of the superimposed signals with the previously determined DEP signals and fixing the EO electrical potential to 2 Vpp. The dashed and dotted horizontal lines indicate positive and negative control values, respectively.

First, the AC electrical potentials and frequencies for DEP and EO of the particles were determined. To determine these values for the optimal DEP-capture of the bacteria against the beads, the real parts of the Clausius–Mossotti (CM) factors for the bacteria and beads were plotted with respect to the AC frequency^37^ (Fig. S1a). The optimal EO frequency for 0.01× phosphate buffered saline (PBS) was also determined by measuring its electrical conductivity and by using the reported Debye length^10^ (Fig. S1b). For MS2 viruses and cTnI-Ab, no models on their dielectrophoretic responses have been reported on, as would be necessary to determine their CM factors; therefore, their DEP characteristics were experimentally investigated by varying the AC frequency from 8 kHz to 1 MHz for cTnI-Ab and from 10 kHz to 10 MHz for the MS2 viruses, and the frequency providing maximal capture was selected as the optimal DEP frequency^38,39^ (Fig. 2a,b). As the Debye lengths for the salty stock solutions of the MS2 viruses and cTnI-Ab were not available, the optimal EO frequencies for the media were also experimentally determined by varying the EO frequencies of the superimposed signals, fixing the previously determined DEP signals [10 Vpp (peak-to-peak)], and finding the intermediate frequencies inducing maximal capture with low electric potentials (2 Vpp)^17^ (Fig. 2c,d). Here, sharp changes in the fluorescence intensities owing to captured viruses and proteins were observed over narrow and low frequency ranges (500 to 2000 Hz and 300 to 800 Hz for viruses and proteins, respectively), which is typical of EO spectra rather than DEP as DEP behavior generally changes with wider frequency ranges^40,41^. Table 2 shows the obtained AC electrical potentials and frequencies for the particles and media.

**Table 2:** Experimental properties and parameters of the particles and media used in this study.

	Bacteria-bead mixture	Virus solution	Protein solution
Particle	*E. coli* K-12 (~0.5 μm wide and ~2 μm long) & 1-μm-diam. PS beads	MS2 bacteriophage (23–28 nm in diameter)^60^	Troponin I antibody (30 kDa)
Media conductivity (measured at 22.9 °C, μS/cm)	184	4	16
Debye length^61^	7.61 nm (0.01x PBS)	—	—
AC frequency (EO)	1633 Hz	1000 Hz	500 Hz
AC electrical potential (EO)	1.2 Vpp	2 Vpp	2 Vpp
AC frequency (DEP)	2 MHz	100 kHz	10 kHz
AC electrical potential (DEP)	5 Vpp	10 Vpp	10 Vpp

The used cTnI-Ab had a weak fluorescence due to their small size (30 kDa), so camera exposure time was determined to be 2 s to enhance the fluorescence images. Exposure time of 1–9 s were reported for other proteins^42,43^ to enhance the measured signal. Moreover, the protein concentration was kept to 500 ng/ml, under which fluorescence images were not clear, and few clumps of the protein were unavoidable in the solution. The vortex flows of the clumps were occasionally observed near the electrodes when using the lower frequency (EO) and superimposed (EO + DEP) signals (see video in the Supplementary Information), but those were not observed when using the higher frequency (DEP).

AC electrothermal (ET) flows can also be considered an alternative to AC EO flows; however, they are commonly induced by Joule heating through salty media or by heating the substrate. AC ET flow is usually dominant at high frequencies (on the order of MHz) or high electrical conductivities (>1000 μS/cm), especially if the applied electric potential is high^27,29,44^. In the present study, AC ET flow was negligible because low conductivity media were used with relatively low electric potentials and no heat sources. In fact, the maximum measured ET flow velocity owing to Joule heating was reported to be ~7 μm/s under the applied electric potentials of 10 Vpp (at 200 kHz) and electrical conductivity of 10000 μS/cm on 60 μm gap coplanar electrodes^45^, which was considerably smaller than the measured flow velocities (~107 μm/s and ~135 μm/s for the bacteria and beads respectively) around the facing electrode edges due to AC EO in the present study. These flow velocities were calculated by measuring the moving distances of the particles around the facing electrode edges during time intervals between two frames of the recorded videos. Furthermore, positive DEP (pDEP) with low conductivity media is stronger and easier to use for particle trapping than negative DEP (nDEP) with high conductivity media^46^. However, it should also be noted that most of biological functionalities are not designed for low conductivity media, and pDEP and EO tend to drop in high conductivity media^46^; hence pDEP and EO can be limited for certain biological applications.

**Figure 3:**
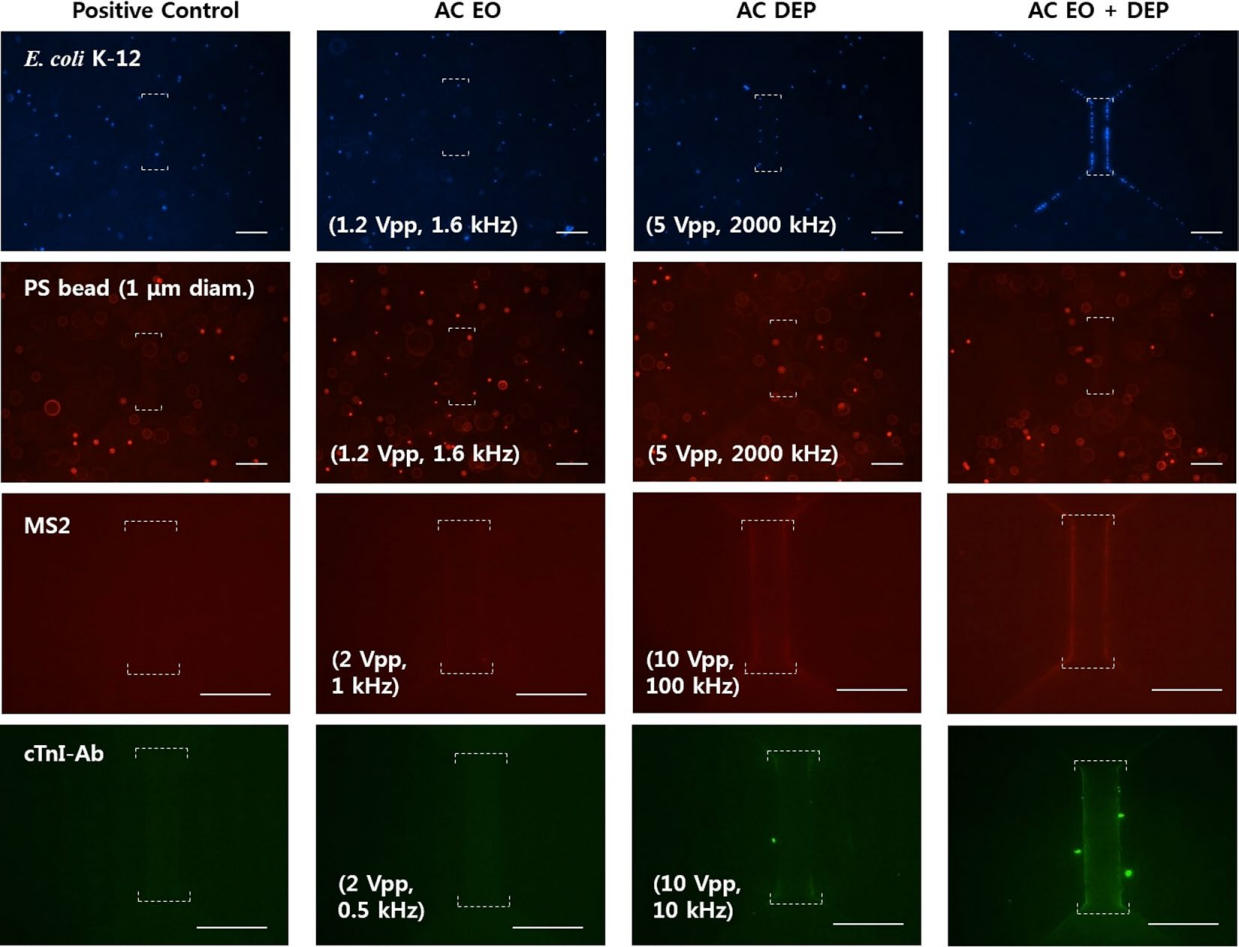
Fluorescence images of the concentrated particles after biasing different electrical signals for 30 s (*Escherichia coli* K-12 and polystyrene beads) and 1 min (MS2 viruses and cTnI-Ab). Different types of electrical signals include no signal (positive control), low frequency signals for EO (1.2 Vpp, 1633 Hz for bacteria-bead; 2 Vpp, 1000 Hz for MS2 virus; 2 Vpp, 500 Hz for cTnI-Ab), high frequency signals for DEP (5 Vpp, 2 MHz for bacteria-bead; 10 Vpp, 100 kHz for MS2 virus; 10 Vpp, 10 kHz for cTnI-Ab), and superimposed signals for EO + DEP. The white scale bars represent 50 μm, and the white dashed lines indicate the top and bottom edges of the rectangular RoIs.

Using the obtained DEP and EO conditions, electrokinetic concentration experiments were conducted for the prepared biological particle solutions with single sinusoidal signals (either DEP or EO) and superimposed signals (DEP + EO). Figure 3 shows fluorescence images of the particles concentrated using different treatments. The superimposed signals concentrated *Escherichia coli* K-12, MS2 viruses, and cTnI-Ab more effectively than the single treatments, as demonstrated by the shiny lines on the facing edges of the electrodes. Regarding the bead experiments, nDEP occurred at the tested frequency, so the beads were not captured in the region of interest (RoI). We also observed noticeable particle movement under the superimposed signals in the videos, which was not observed under DEP bias only.

**Figure 4:**
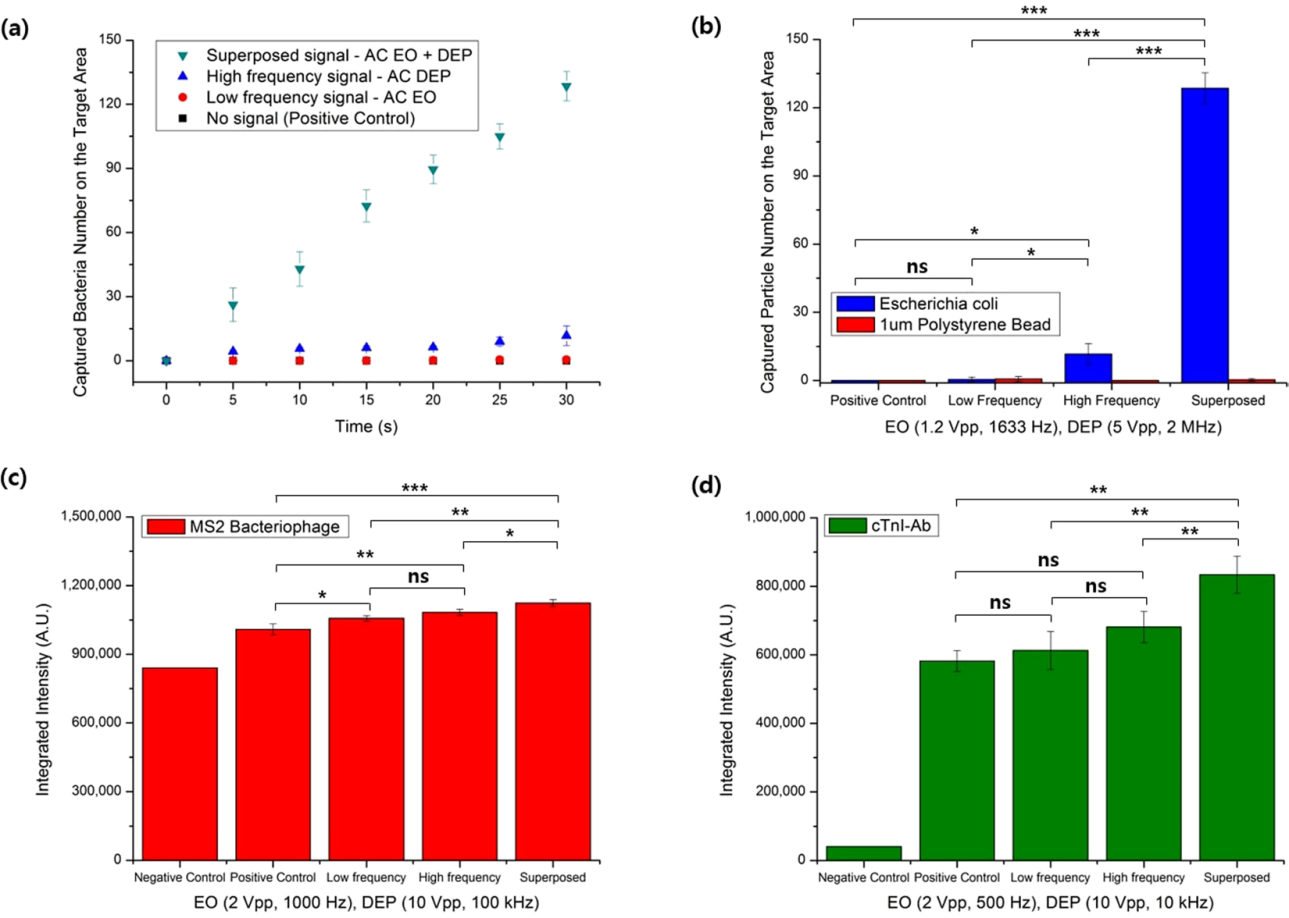
Amounts of particles collected within the RoIs using different electrical signals. (**a**) *Escherichia coli* K-12 collection over time^36^. (**b**) *E. coli* K-12 and beads collected. (**c**) Integrated intensities of concentrated MS2 viruses. (**d**) Integrated intensities of collected cTnI-Ab. The experiments were conducted for 30 s (*E. coli* K-12 and polystyrene beads) and 1 min (MS2 viruses and cTnI-Ab) using different electrical signals: no solution addition (negative control), no signal after adding the solution (positive control), low frequency EO signals, high frequency DEP signals, and superimposed signals (EO + DEP). Statistical analysis was performed using one-way ANOVA followed by the Tukey post hoc test, and statistically significant results are identified with asterisks (***, and ***p values < 0.05, 0.01 and 0.0001, respectively; ns – not significant).

Figure 4a shows the numbers of *E. coli* K-12 collected within the RoI over time for different electrical signals. More of the bacteria were captured using the superimposed signals than any of the single electrical signals. In fact, the numbers of *E. coli* K-12 captured by EO and DEP alone are 0.4% and 9.1%, respectively, of the number collected in the superimposed signal case at 30 s. This result can be ascribed to the fact that DEP is usually effective for the particles close to the electrode surface, and EO flow can drag particles over the electrode toward the region between the electrodes without capturing most of them. The bacterial capture with EO alone in this study was not significantly different from that with no signal treatment (*p* = 0.999), whereas the bacterial capture with DEP alone was significantly different from that with no signal treatment (*p* = 0.034). By contrast, the bacterial capture with the superimposed signals was significantly larger than those with the other two electrical treatments. The superimposed signals provided the advantages of both DEP and EO, first moving distant particles toward the region between the electrodes with EO flow, and then capturing the moved particles at a particular position, where the largest electric field occurs, against the flow with pDEP. This superimposition can be employed to enhance the sensor sensitivity and reduce the detection time when used with biosensors^18^. In fact, the amount of DEP-assisted attachment of cTnI (cardiac troponin I) after 1 min was less than that due to sedimentation for 1 h^18^ in a cTnI sensor, because the proteins far from the electrode might not be attracted toward the electrode rapidly with DEP alone.

Figure 4b shows the numbers of bacteria and beads captured after applying different treatments for 30 s. A moderate speed of ~119 (±6.3) μm/s around the facing electrode edges was adopted for the superimposed treatments, because it allowed many bacteria over the electrodes to be collected by pDEP while the beads were repelled from the electrode edges by nDEP without inertial attachment to the surface, making it possible to collect the bacteria selectively from the bacteria–bead mixture (Supplementary Video 1).

**Figure 5:**
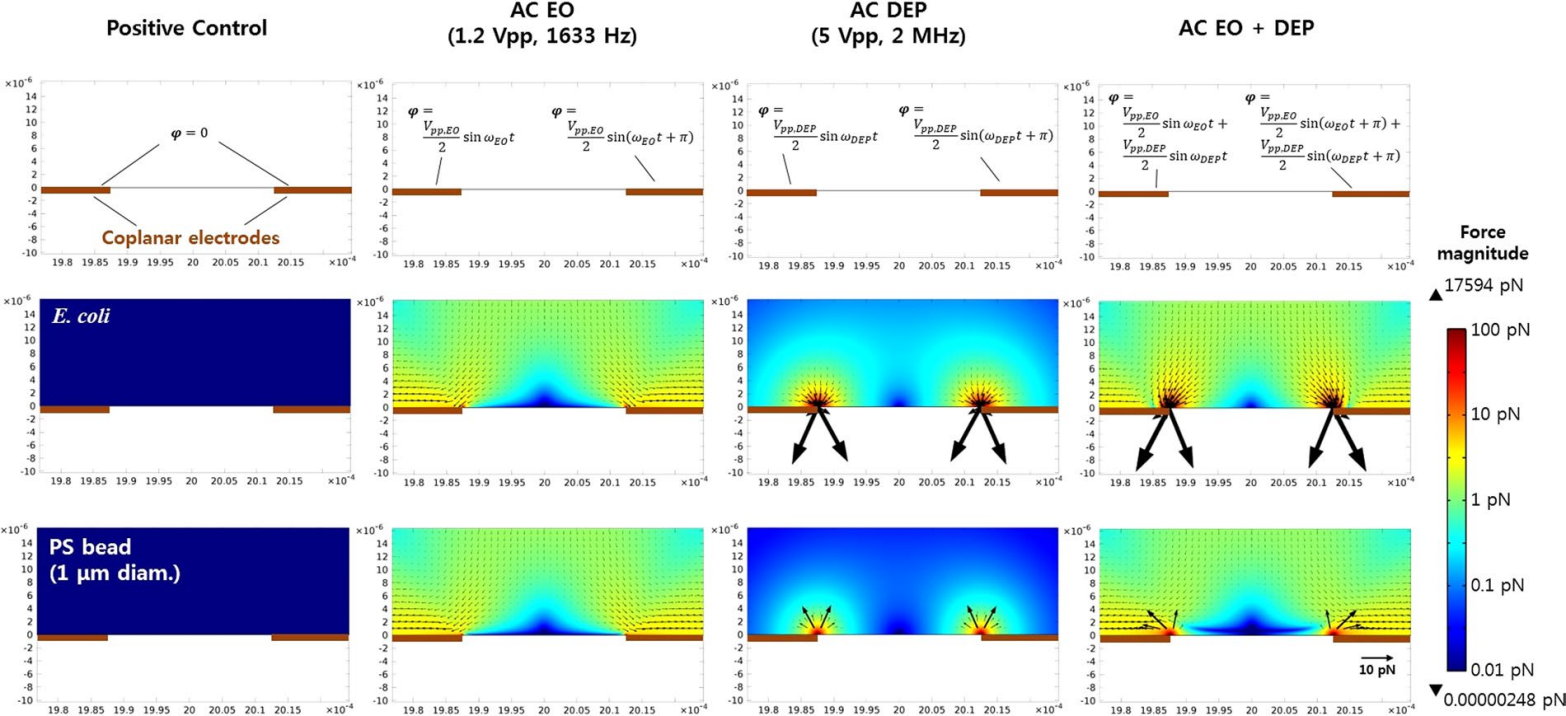
Calculated net force fields for *E. coli* and 1 μm-diam. PS beads that are initially at rest under different electrical treatments. Each surface plot represents the magnitude of the resultant forces exerted on the particles, and the black arrows show the force vectors.

Figure 5 shows the calculated net force fields for *E. coli* and PS beads using COMSOL Multiphysics® 4.3, and hydrodynamic drag, gravitational, buoyant, and DEP forces were considered for the force field calculation (Supplementary Information). The simulation was conducted for actual experimental geometry (Fig. S2), but close views around the electrodes were demonstrated here to show the differences between the treatments effectively. DEP (pDEP for bacteria and nDEP for beads) was effective within several microns from the electrode edges, and AC-EO dragged flows containing the particles to the electrode edges. The superimposed signal shows the integrated effect of DEP and EO for the enhanced selective concentration of the bacteria against beads (Supplementary Video 2).

The purity of the concentrated bacteria against the beads, i.e., the separation efficiency, was kept more than 99%, where the separation efficiency was defined as the fraction of target particles with respect to all of the particles (target + non-target). High separation efficiencies (over 90%) by DEP for bacterial capture were previously reported using applied voltages of more than 20 Vpp and long electrical activation times (10 min–1 h)^47–49^. The superimposed signals enhanced the bacterial capture more selectively (>99%) and more rapidly (30 s) with a low electric potential of DEP (5 Vpp) and simultaneous use of EO (1.2 Vpp).

Figure 4c,d show the quantities of MS2 viruses and cTnI-Ab, respectively, that were collected after applying the different types of signals for 1 min. Although nanometer-sized particles such as viruses and proteins are known to be difficult to manipulate by using DEP due to their small sizes, several studies have demonstrated the successful use of DEP for these particles^50,51^. In those studies, long exposure times (5–30 min)^52–54^ or nanoscale electrode gaps (30–500 nm)^38,39,55^ under applied voltages of 5–35 Vpp were applied to increase the electric field for capture. It was observed in the present study that significantly more MS2 and cTnI-Ab were captured using the superimposed signals than either DEP (10 Vpp) or EO (2 Vpp) signals alone (*p* < 0.035), with a gap of 25 μm between two electrodes and short electric field exposure time (1 min). The electric field gradient in the present electrodes was not high, compared to the previous DEP studies; however, many of the nanoparticles in the present study were continuously moved to the electrode edges by AC-EO, providing those nanoparticles with the chances to be affected by the DEP forces. That is, applying AC-EO corresponded to increasing virus concentration near the electrodes. This was made possible by controlling the voltages and frequencies of DEP and EO separately, thereby precluding electrolysis as well as increasing the electric field intensity for capture.

## Conclusions

We have demonstrated the rapid and selective electrokinetic concentration of bacteria, viruses, and proteins on two coplanar electrodes via superimposition of AC EO with DEP. The superimposed signals moved the particles distant from the electrodes toward the high electric field area with EO irrespective of the particle size, and then captured the particles selectively at a particular position, i.e., the highest electric field spot, against the flow with pDEP. Significantly more bacteria were captured using the superimposed signals than were collected using the other two treatments, EO and DEP. Moreover, the bacteria were selectively and rapidly concentrated with high purity against polystyrene beads from the mixture. The concentrations of collected nanometer-sized biological particles such as MS2 viruses and cTnI-Ab proteins were also enhanced by using this superimposition (EO + DEP) technique. This technique allowed for a relatively large gap between two electrodes and short electric field exposure time, and high capture efficiency. We believe that the superimposition of AC EO and DEP can be applied to many biosensors requiring the rapid detection of biological particles with simple coplanar electrodes^17,18^.

## Materials and Methods

### Materials

The following materials were purchased from commercial sources: 4′,6-diamidino-2-phenylindole (DAPI; D9564), rhodamine B (Rh-B; R6626), dialysis tubing (D0405-100FT; molecular weight cut-off: 12400), *N*-hydroxysuccinimide (NHS; 130672), N-(3-dimethylaminopropyl)-*N*′-ethylcarbodiimidehydrochloride (EDC; 03449) from Sigma-Aldrich (USA); polydimethylsiloxane (PDMS; Sylgard® 184) from Dow Corning Corp. (USA); sterile Acrodisc® syringe filters with Supor® membrane (4612; pore size: 0.2 μm) from Pall Corporation (USA); phosphate buffered saline (PBS; 20×, pH 7.4) from Biosesang Inc. (Korea); Luria-Bertani broth (LB broth; 244620) and tryptic soy broth (TSB; 211825) from Becton, Dickinson and Company (USA); fluorescent PS beads (Fluoro-Max R0100; diameter: 1 μm) from Thermo Scientific (USA); *Escherichia coli* K-12 (ATCC® 25404™), *Escherichia coli* C3000 (ATCC® 15597™), and MS2 bacteriophages (ATCC® 15597-B1™) from PLS (Korea); and FITC-linked polyclonal antibody to troponin I type 3, cardiac (TNNI3) (LAA478Mu81) from Cloud-Clone Corp. (USA). Distilled water (18.2 MΩ∙cm) was obtained through university-established water pipelines.

### Microfabrication of Chips and Experimental Set-up

Two 100-nm-thick indium tin oxide (ITO) coplanar electrodes were fabricated on a glass wafer (6 in. diameter) with 25 μm gaps, using the conventional photolithography and radio-frequency sputtering. The ITO electrodes were then annealed for 1 h at 400 °C in an oven for transparency and electrical resistance reduction. The wafer was diced into chips (1 × 1 cm^2^) with two coplanar electrodes on each chip, which are shown in the bright field image in Fig. 1a. The detailed fabrication procedure is in the Supplementary Information. An inverted microscope (Eclipse Ti-U; Nikon, Japan) was used to observe the particle motion around the electrodes, maintaining the optical focus on the transparent electrode surface. A 30 μl PDMS well was located at the center of the chip, and 20 μl of the prepared solution was added into the well. Different electrical signals were then applied to the ITO electrodes for either 30 s (for the bacteria–beads) or 1 min (for the viruses and proteins).

Two dual-channel arbitrary function generators (AFG3022C; Tektronix, USA) were used to generate sinusoidal signals 180° out of phase. Four signals from the two function generators were superimposed by a lab-made voltage adder consisting of impedance buffers and frequency mixers, and the signals were monitored by an oscilloscope (DS2072A; RIGOL Technologies Inc., USA) (Fig. 1b).

Videos and images of concentrated fluorescent particles were taken by a cooled interline transfer charge-coupled device camera (ORCA-R2; Hamamatsu, Japan), and the quantities of particles collected in the fixed regions of interests (RoI; 120 × 360 pixels) between the two ITO electrodes were measured using ImageJ. The numbers of particles in the RoI were determined by dividing the total particle area by the single particle area for the bacteria and beads^56^ and by measuring the integrated intensities for the viruses and proteins^38,55^. The exposure times for fluorescence imaging and video recordings were 100 ms, 40 ms, 500 ms, and 2 s for the bacteria, beads, viruses, and proteins, respectively, and the videos were recorded using the maximal frame per second setting for each experiment.

### Preparation of Biological Particle Solutions with Fluorescence Labeling

Three types of biological particle solutions, bacteria–bead mixtures, viruses, and proteins, were prepared. For the bacteria, 10 μl of *E. coli* K-12 stock was added to 10 ml of LB broth solution, and the bacteria were grown at 37 °C and 160 rpm in a shaking incubator for 12 h. They were centrifuged at 4000 rpm for 10 min to remove the residual LB broth. The remaining sunk bacteria were suspended in DI water for DAPI labeling (excitation/emission: 360/460 nm) to distinguish them from the red fluorescent beads. The labeled bacteria were then centrifuged and re-suspended in 0.01× PBS^57^. The bacterial number concentration was determined by performing optical density measurements at 600 nm^58^, and the final bacteria concentration was 1 × 10^7^/ml. Red fluorescent (excitation/emission: 542/612 nm) PS beads 1 μm in diameter were suspended in 0.01× PBS buffer with a number density of 1 × 10^7^/ml, and the bacteria and bead solutions were mixed.

For the virus experiments, freeze-dried MS2 phages were dissolved in 1× PBS to obtain a viral mass concentration of 1 mg/ml. Then, 0.5 ml of the MS2 solution was added to 10 ml of *E. coli* C3000, the host bacterium for MS2 bacteriophages, and incubated at 37 °C and 160 rpm for 5 h. The mixture was then centrifuged at 3000 rpm for 10 min to remove the bacteria, and the MS2-laden supernatant was filtered using a membrane filter. The prepared MS2 stock was then labeled with red fluorescence dye Rh-B (excitation/emission: 562/583 nm in water) by coupling EDC and NHS, and the stock and dye were mixed and purified in a dialysis membrane for 1 week to remove the unbound dye^59^. The concentration of the labeled virus stock solution was approximately 10^7^ plaque forming units (pfu)/ml, which was verified by a plaque assay, and the solution was 10,000-fold diluted in DI water. For the protein experiments, FITC-labelled cTnI-Ab was used (excitation/emission: 495/525 nm), and its stock solution was 400-fold diluted in DI water for a mass concentration of 500 ng/ml. The media conductivities of all three test solutions were measured using a conductivity meter (handylab pH/LF 12; SI Analytics GmbH, Germany) (Table 2).

### Statistical Analysis

Each experiment in this study was performed at least three times. The average values are shown in the figures with their standard deviations indicated as error bars. Statistical analysis was performed using one-way analysis of variance (ANOVA) followed by the Tukey post hoc test (Table S1). Significantly different results (*p* < 0.05, 0.01, and 0.0001) are designated with asterisks (***, and ***, respectively).

## Electronic supplementary material

Supplementary Information

Supplementary Video 1

Supplementary Video 2

## References

[1] Pohl HA (1951). The motion and precipitation of suspensions in divergent electric fields. J. Appl. Phys..

[2] Yang J, Huang Y, Wang X-B, Becker FF, Gascoyne PRC (2000). Differential analysis of human leukocytes by dielectrophoretic field-flow-fractionation. Biophys. J..

[3] Becker FF (1995). Separation of human breast cancer cells from blood by differential dielectric affinity. Proc. Natl. Acad. Sci. USA.

[4] Wang X–B, Vykoukal J, Becker FF, Gascoyne PRC (1998). Separation of polystyrene microbeads using dielectrophoretic/gravitational field-flow-fractionation. Biophys. J..

[5] Li H, Bashir R (2002). Dielectrophoretic separation and manipulation of live and heat-treated cells of Listeria on microfabricated devices with interdigitated electrodes. Sens. Actuators B.

[6] Morgan H, Hughes MP, Green NG (1999). Separation of submicron bioparticles by dielectrophoresis. Biophys. J..

[7] Green NG, Ramos A, Morgan H (2002). Numerical solution of the dielectrophoretic and travelling wave forces for interdigitated electrode arrays using the finite element method. J. Electrostat..

[8] Markx GH, Pethig R (1995). Dielectrophoretic separation of cells: continuous separation. Biotechnol. Bioeng..

[9] Gadish N, Voldman J (2006). High-throughput positive dielectrophoretic bioparticle microconcentrator. Anal. Chem..

[10] Ramos A, Morgan H, Green NG, Castellanos A (1999). AC electric-field-induced fluid flow in microelectrodes. J. Colloid Interface Sci..

[11] Brown ADB, Smith CG, Rennie AR (2000). Pumping of water with ac electric fields applied to asymmetric pairs of microelectrodes. Phys. Rev. E.

[12] Studer V, Pepin A, Chen Y, Ajdari A (2004). An integrated AC electrokinetic pump in a microfluidic loop for fast and tunable flow control. Analyst.

[13] Huang C-C, Bazant MZ, Thorsen T (2010). Ultrafast high-pressure AC electro-osmotic pumps for portable biomedical microfluidics. Lab Chip.

[14] Sasaki N, Kitamori T, Kim H-B (2006). AC electroosmotic micromixer for chemical processing in a microchannel. Lab Chip.

[15] Chen J-K, Weng C-N, Yang R-J (2009). Assessment of three AC electroosmotic flow protocols for mixing in microfluidic channel. Lab Chip.

[16] Chen J-L, Shih W-H, Hsieh W-H (2013). AC electro-osmotic micromixer using a face-to-face, asymmetric pair of planar electrodes. Sens. Actuators B: Chem..

[17] Gong J-R (2010). Label-free attomolar detection of proteins using integrated nanoelectronic and electrokinetic devices. Small.

[18] Sharma A, Han C-H, Jang J (2016). Rapid electrical immunoassay of the cardiac biomarker troponin I through dielectrophoretic concentration using imbedded electrodes. Biosens. Bioelectron..

[19] Heeren A, Luo CP, Henschel W, Fleischer M, Kern DP (2007). Manipulation of micro- and nano-particles by electro-osmosis and dielectrophoresis. Microelectron. Eng..

[20] Cheng I-F, Chang H-C, Chen T-Y, Hu C, Yang F-L (2013). Rapid (<5 min) identification of pathogen in human blood by electrokinetic concentration and surface-enhanced raman spectroscopy. Sci. Rep..

[21] Gagnon Z, Chang H-C (2005). Aligning fast alternating current electroosmotic flow fields and characteristic frequencies with dielectrophoretic traps to achieve rapid bacteria detection. Electrophoresis.

[22] Zhou H, White LR, Tilton RD (2005). Lateral separation of colloids or cells by dielectrophoresis augmented by AC electroosmosis. J. Colloid Interface Sci..

[23] Lin S-C, Tung Y-C, Lin C-T (2016). A frequency-control particle separation device based on resultant effects of electroosmosis and dielectrophoresis. Appl. Phys. Lett..

[24] Rezanoor MW, Dutta P (2016). Combined AC electroosmosis and dielectrophoresis for controlled rotation of microparticles. Biomicrofluidics.

[25] Melvin EM, Moore BR, Gilchrist KH, Grego S, Velev OD (2011). On-chip collection of particles and cells by AC electroosmotic pumping and dielectrophoresis using asymmetric microelectrodes. Biomicrofluidics.

[26] Green NG, Ramos A, Gonzalez A, Castellanos A, Morgan H (2000). Electric field induced fluid flow on microelectrodes: the effect of illumination. J. Phys. D: Appl. Phys..

[27] Park S, Koklu M, Beskok A (2009). Particle trapping in high-conductivity media with electrothermally enhanced negative dielectrophoresis. Anal. Chem..

[28] Sang S (2016). Portable microsystem integrates multifunctional dielectrophoresis manipulations and a surface stress biosensor to detect red blood cells for hemolytic anemia. Sci. Rep..

[29] Castellanos A, Ramos A, Gonzalez A, Green NG, Morgan H (2003). Electrohydrodynamics and dielectrophoresis in microsystems: scaling laws. J. Phys. D: Appl. Phys..

[30] Pethig R, Talary MS, Lee RS (2003). Enhancing traveling-wave dielectrophoresis with signal superposition. IEEE Eng. Med. Biol. Mag..

[31] Valero A, Braschler T, Demierre N, Renaud PA (2010). Miniaturized continuous dielectrophoretic cell sorter and its applications. Biomicrofluidics.

[32] Han S-I, Joo Y-D, Han K-H (2013). An electrorotation technique for measuring the dielectric properties of cells with simultaneous use of negative quadrupolar dielectrophoresis and electrorotation. Analyst.

[33] Cui H-H, Voldman J, He X-F, Lim K-M (2009). Separation of particles by pulsed dielectrophoresis. Lab Chip.

[34] Kumemura M (2011). Single-DNA-molecule trapping with silicon nanotweezers using pulsed dielectrophoresis. J. Micromech. Microeng..

[35] Honegger T, Peyrade D (2013). Moving pulsed dielectrophoresis. Lab Chip.

[36] Han, C.-H., Woo, S. Y., Sharma, A., Jang, J. Rapid and selective electrokinetic concentration of bacteria using AC signal superposition on two coplanar electrodes. In proceedings of the 21st international conference on miniaturized systems for chemistry and life sciences (MicroTAS 2017), Oct 22-26, 1315–1317 (2017).

[37] Park S, Zhang Y, Wang T-H, Yang S (2011). Continuous dielectrophoretic bacterial separation and concentration from physiological media of high conductivity. Lab. Chip..

[38] Madiyar FR, Syed LU, Culbertson CT, Li J (2013). Manipulation of bacteriophages with dielectrophoresis on carbon nanofiber nanoelectrode arrays. Electrophoresis.

[39] Hölzel R, Calander N, Chiragwandi Z, Willander M, Bier FF (2005). Trapping single molecules by dielectrophoresis. Phys. Rev. Lett..

[40] Morgan H, Green NG (1997). Dielectrophoretic manipulation of rod-shaped viral particles. J. Electrostat..

[41] Camacho-Alanis F, Ros A (2015). Protein dielectrophoresis and the link to dielectric properties. Bioanalysis.

[42] Zhou J, Lin J, Zhou C, Deng X, Xia B (2011). Cytotoxicity of red fluorescent protein DsRed is associated with the suppression of Bcl-xL translation. FEBS Lett..

[43] Williams EH (2013). Selective streptavidin bioconjugation on silicon and silicon carbide nanowires for biosensor applications. J. Mater. Res..

[44] Oh J, Hart R, Capurro J, Noh H (2009). Comprehensive analysis of particle motion under non-uniform AC electric fields in a microchannel. Lab Chip.

[45] Feldman HC, Sigurdson M, Meinhart CD (2007). AC electrothermal enhancement of heterogeneous assays in microfluidics. Lab Chip.

[46] Voldman J (2006). Dielectrophoretic traps for cell manipulation. BioMEMS Biomed. Nanotechnol..

[47] Cheng I-F, Froude VE, Zhu Y, Chang H-C, Chang H-C (2009). A continuous high-throughput bioparticle sorter based on 3D traveling-wave dielectrophoresis. Lab Chip.

[48] Kim U, Soh HT (2009). Simultaneous sorting of multiple bacterial targets using integrated dielectrophoretic-magnetic activated cell sorter. Lab Chip.

[49] Elitas M, Martinez-Duarte R, Dhar N, McKinney JD, Renaud P (2014). Dielectrophoresis-based purification of antibiotic-treated bacterial subpopulations. Lab Chip.

[50] Nakano A, Ros A (2013). Protein dielectrophoresis: advances, challenges, and applications. Electrophoresis.

[51] Dash S, Mohanty S (2014). Dielectrophoretic separation of micron and submicron particles: a review. Electrophoresis.

[52] Green NG, Morgan H, Milner JJ (1997). Manipulation and trapping of sub-micron bioparticles using dielectrophoresis. J. Biochem. Biophys. Methods.

[53] Hughes MP, Morgan H (1998). Dielectrophoretic trapping of single sub-micrometre scale bioparticles. J. Phys. D: Appl. Phys..

[54] Maruyama H (2011). Nanomanipulation of single influenza virus using dielectrophoretic concentration and optical tweezers for single virus infection to a specific cell on a microfluidic chip. Microfluid. Nanofluid.

[55] Liao K-T, Chou C-F (2012). Nanoscale molecular traps and dams for ultrafast protein enrichment in high-conductivity buffers. J. Am. Chem. Soc..

[56] Yang L (2006). A multifunctional micro-fluidic system for dielectrophoretic concentration coupled with immune-capture of low numbers of Listeria monocytogenes. Lab Chip.

[57] Lapizco-Encinas BH, Simmons BA, Cummings EB, Fintschenko Y (2004). Dielectrophoretic concentration and separation of live and dead bacteria in an array of insulators. Anal. Chem..

[58] Ausubel, F. M. *et al*. *Current Protocols in Molecular Biology* (John Wiley & Sons, Inc., 2003).

[59] Gitis V, Adin A, Nasser A, Gun J, Lev O (2002). Fluorescent dye labeled bacteriophages – a new tracer for the investigation of viral transport in porous media: 1. introduction and characterization. Water Res..

[60] Kuzmanovic DA, Elashvili I, Wick C, O’Connell C, Krueger S (2003). Bacteriaphage MS2: molecular weight and spatial distribution of the protein and RNA components by small-angle neutron scattering and virus counting. Structure.

[61] Manchester, M., Steinmetz, N. F. *Viruses and Nanotechnology* (Springer-Verlag, 2009).

