# Supplementary material for: Rapid and selective concentration of bacteria, viruses, and proteins using alternating current signal superimposition on two coplanar electrodes
Source: Sci Rep. 2018 Oct 8;8:14942. doi: 10.1038/s41598-018-33329-7 (PMC6175930; doi:10.1038/s41598-018-33329-7)
Supplement: Supplementary file 1 — Supplementary Information [file 41598_2018_33329_MOESM1_ESM.pdf]

Supplementary Information

Rapid and selective concentration of bacteria, viruses, and proteins using alternating current signal superimposition on two coplanar electrodes

Chang-Ho Han[a], Seong Yong Woo[a], Jyoti Bhardwaj[b], Abhinav Sharma[c], Jaesung Jang[a,b,†]

[a] School of Mechanical, Aerospace and Nuclear Engineering, Ulsan National Institute of Science and Technology (UNIST), Ulsan 44919, Republic of Korea

[b] Department of Biomedical Engineering, UNIST, Ulsan 44919, Republic of Korea

[c] School of Materials Science and Engineering, UNIST, Ulsan 44919, Republic of Korea

[†]Correspondence should be addressed to jjang@unist.ac.kr; Tel: +82-52-217-2323; Fax: +82-52-217-2449

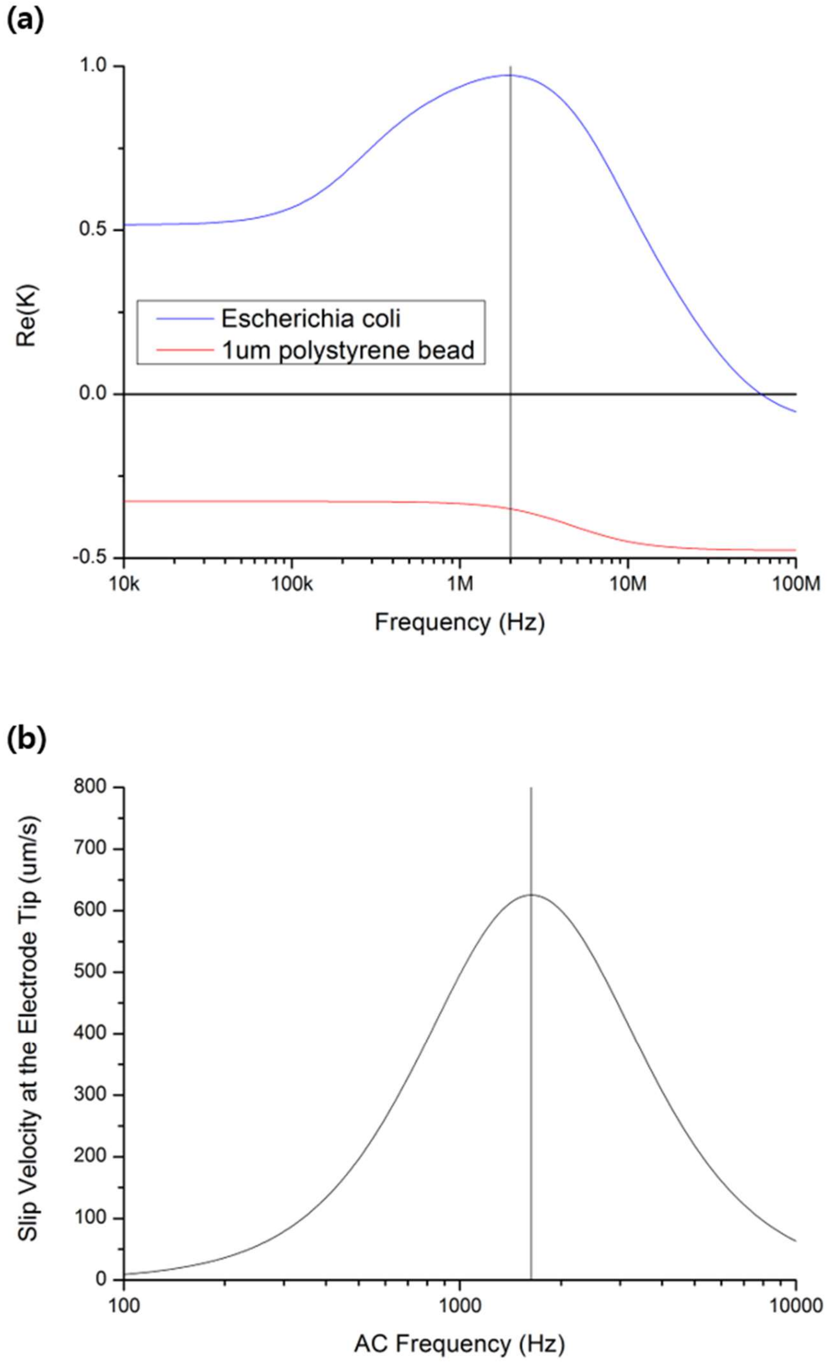

Figure S-1. (a) Calculated real parts of the Clausius–Mossotti factors for *Escherichia coli* K-12 and 1-μm-diam. polystyrene beads, suspended in 0.01× phosphate buffered saline (PBS) (184 μS/cm) [1]. **(b)** Calculated slip velocity magnitude at the electrode tip in 0.01× PBS buffer with an applied electrical potential of 1.2 Vpp.

Theoretical backgrounds

The time-averaged dielectrophoretic force exerted on a particle suspended in a medium of electrical permittivity ε_m under a non-uniform electric field is expressed as [2–4]

$$\overline{F}_{DEP} = 2\pi\varepsilon_m {r_p}^3 Re[f_{CM}(\omega)]\underline{\nabla}\left|\overline{E}_{rms}\right|^2, \qquad \text{(eq. 1)}$$

where r_p is the radius of the particle, $Re[f_{CM}(\omega)]$ is the real part of the Clausius-Mossotti (CM) factor depending on the angular frequency (rad/s), ω, of AC signals, and $\overline{E}_{rms}$ is the root-mean-squared electric field intensity vector. When the CM factor is not available for a particle suspended in a medium, the optimal magnitude and frequency of the dielectrophoretic force can be experimentally determined by varying the AC frequency.

Regarding the optimal electroosmosis (EO) conditions, when slip flow is induced on the coplanar electrodes due to AC EO, the time-averaged velocity can be expressed as, under an assumption of negligible Stern layer effects [5–7],

$$\langle v_{ACEO}\rangle = \frac{\varepsilon_m {\varphi_0}^2 \Omega^2}{8\mu x(1+\Omega^2)^2}, \qquad \text{(eq. 2)}$$

$$\Omega = \frac{\pi x \varepsilon_m \omega}{2\sigma_m \lambda_D}, \qquad \text{(eq. 3)}$$

where φ_0 is the initial potential, Ω is a non-dimensional frequency, μ is the dynamic viscosity, x is the position starting from the center of the gap between the two coplanar electrodes, σ_m is the electrical conductivity of media, and λ_D is a Debye length. The optimal AC frequency is one that provides the highest $\langle v_{ACEO}\rangle$.

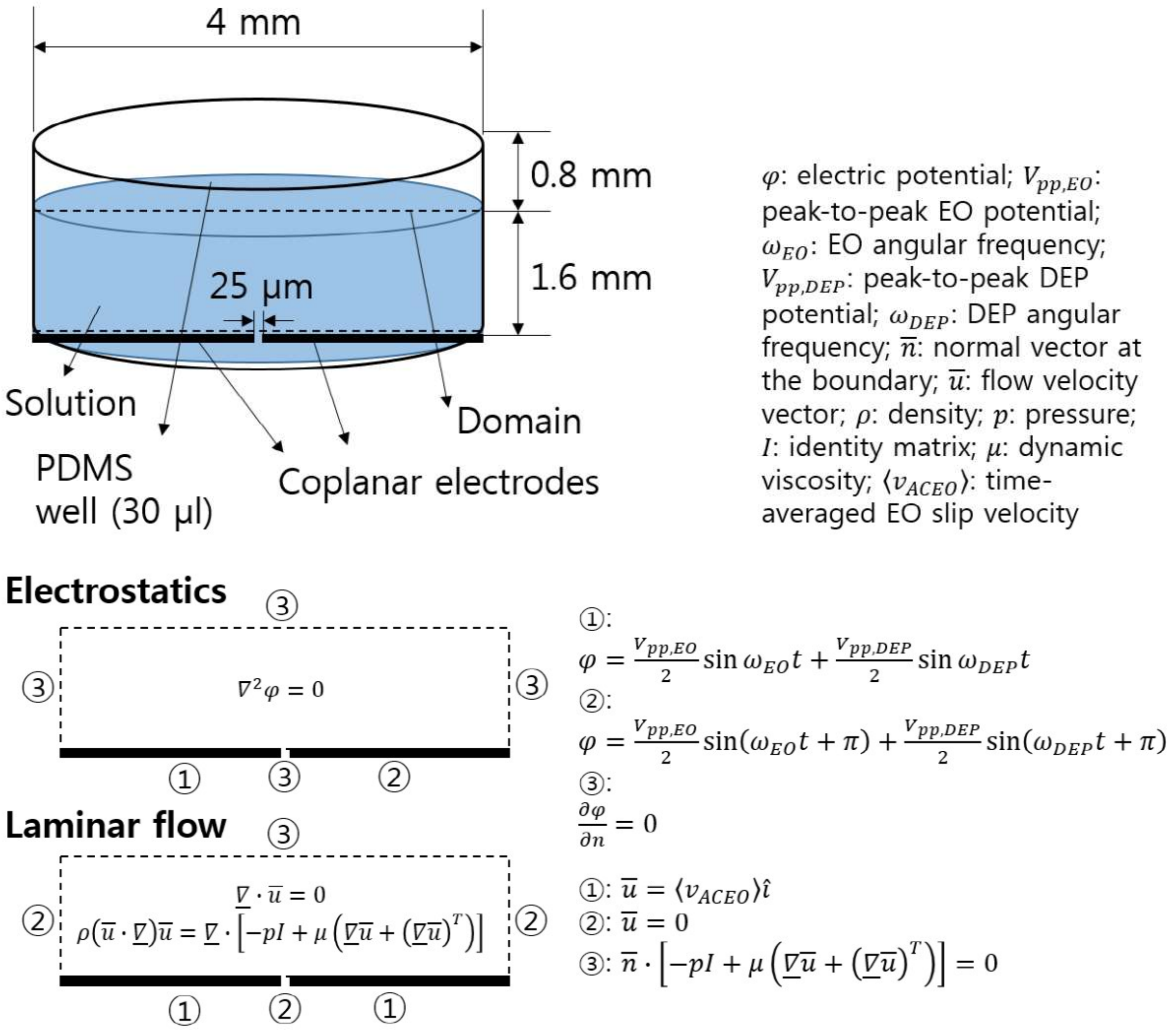

Figure S-2. Problem formulation for the electrokinetic simulation.

Electrokinetics simulation using finite element method and particle traces

2D simulation was conducted using a commercial software COMSOL Multiphysics® 4.3. A 4 mm long (x-) and 1.6 mm high (y-) rectangular domain was designed according to the experiments. Quasi-static electric potential and laminar flow fields were calculated with the governing equations and boundary conditions (Fig. S-2) [6]. The net force fields exerted on the particles were calculated along the domain, with the Newtonian force model, which is given by

$$m_p \frac{d^2\bar{x}_p}{dt^2} = \bar{F}_{drag} + \bar{F}_{gravity} + \bar{F}_{buoyancy} + \bar{F}_{DEP} = -3\pi\mu d_p\left(\frac{d\bar{x}_p}{dt} - \bar{u}\right) + m_p \frac{\rho_p-\rho}{\rho_p}\bar{g} + \frac{\pi}{4}{d_p}^3\varepsilon_m Re(K)\underline{\nabla}\left|\bar{E}\right|^2 = F_x\hat{\imath} + F_y\hat{\jmath},$$

where m_p is the particle mass, $\bar{x}_p$ is the position vector of particle, d_p is the particle diameter, ρ_p is the particle density, and $\bar{g}$ is the gravity vector. Here, both bacteria and beads were assumed to be 1-μm-diameter spheres, with ρ_p of 1160 [8] and 1050 kg/m^3, respectively, and $Re(K)$ values were considered according to Fig. S-1a. Particles were traced with the time (0 to 30 s) under the same force model (video).

For the electrostatics, multifrontal massively parallel sparse direct solver was used to solve the linear static finite element problems having symmetric matrices for the φ along the entire domain. The converged solutions for $\left|\underline{\nabla}\left|\bar{E}\right|^2\right|$ were obtained for 305,460 elements with 3rd-order elements for the φ (10 nodes per each element). Flow fields were solved using Newton-Raphson algorithms to solve the nonlinear static finite element problems, with an iterative solver (generalized minimum residual method) for the linear systems having nonsymmetric matrices in each step, and hence $\bar{u}$ and p fields were obtained along the domain, where $\bar{u} = u_x\hat{\imath} + u_y\hat{\jmath}$. Convergence of the flow velocity magnitudes was verified at the number of triangular mesh element of 104,217 with 1st-order elements for u_x, u_y, and p (3 nodes per each element).

Particle tracing was conducted using a transient implicit solver (generalized alpha) with automatically scaled time step sizes. 1020 particles were equally distributed at rest ($\frac{d\bar{x}_p}{dt} = 0$), and the predetermined $\bar{u}$ and $\underline{\nabla}\left|\bar{E}\right|^2$ were coupled. Numerical convergence of the particle position was verified at the number of triangular mesh element of 305,460 with the time step between 2.9e-8 to 0.5 s.

Statistical analysis

Statistical analyses comparing the results between four electrical treatments (positive control, AC EO, AC DEP, AC EO+DEP) were performed using one-way analysis of variance (ANOVA) followed by the Tukey post hoc test. Table S-1 shows the obtained *p*-values from the analyses.

Table S-1. Obtained *p*-values from the statistical analyses.

	***p*-value**		
	**Bacteria**	**Viruses**	**Proteins**
**Overall ANOVA**	3.33E-12	1.39E-04	1.11E-04
**Means Comparisons (Tukey post hoc test)**			
**AC EO vs. Positive Control**	0.99867	0.03941	0.9863
**AC DEP vs. Positive Control**	0.0337	6.96E-04	0.13508
**AC DEP vs. AC EO**	0.02982	0.23652	0.23767
**AC EO+DEP vs. Positive Control**	0	2.92E-05	2.01E-04
**AC EO+DEP vs. AC EO**	0	0.0043	3.29E-04
**AC EO+DEP vs. AC DEP**	0	0.03402	0.00238

Microfabrication of the electrodes

The designed coplanar electrodes were fabricated on a glass wafer using the conventional photolithography and radio-frequency (RF) sputtering. First, Borofloat 33 glass wafer (6 in. diameter) was pre-baked on a hot plate at 105 ℃ for 120 s. Adhesion promoter (Hexamethyldisilazane) and positive photoresist (AZ 5214E; Microchemicals GmbH, Germany) was sequentially spin-coated on the wafer at 4000 rpm for 30 s. Soft-baking was then applied on a hot plate at 105 ℃ for 90 s. The baked photoresist layer was exposed to ultraviolet light for 90 mJ/cm^2, and developed within 1 to 2 min.

After patterning the photoresist layer, a 100 nm-thick indium tin oxide (ITO) layer was deposited using RF sputtering, and the ITO electrodes were obtained on glass wafer by the lift-off process in acetone and isopropyl alcohol. The thickness of electrode was measured using a reflectometer. The electrodes were then annealed at 400 ℃ for 1 h in a diffuse furnace for transparency and electrical resistance reduction of ITO. The wafer was diced into chips (1×1 cm^2).

References

[1] Park, S., Zhang, Y., Wang, T.-H, Yang, S. Continuous dielectrophoretic bacterial separation and concentration from physiological media of high conductivity. *Lab. Chip.* **11**, 2893 (2011).

[2] Pohl, H. A. The motion and precipitation of suspensoids in divergent electric fields. *J. Appl. Phys.* **22**, 869-871 (1951).

[3] Pohl, H. A. *Dielectrophoresis* (Cambridge University Press, 1978).

[4] Huang, Y. *et al.* Electric manipulation of bioparticles and macromolecules on microfabricated electrodes. *Anal. Chem.* **73**. 1549-1559 (2001).

[5] Ramos, A., Morgan, H., Green, N. G. Castellanos, A. AC electric-field-induced fluid flow in microelectrodes. *J. Colloid Interface Sci.* **217**, 420-422 (1999).

[6] Oh, J., Hart, R., Capurro, J., Noh, H. Comprehensive analysis of particle motion under non-uniform AC electric fields in a microchannel. *Lab Chip* **9**, 62-78 (2009).

[7] Swaminathan, V. V., Shannon, M. A., Bashir, R., Enhanced sub-micron colloidal particle separation with interdigitated microelectrode arrays using mixed AC/DC dielectrophoretic scheme. *Biomed. Microdevices* **17**, 29 (2015).

[8] Godin, M., Bryan, A. K., Burg, T. P. Measuring the mass, density, and size of particles and cells using a suspended microchannel resonator. *Appl. Phys. Lett.* **91**, 123121 (2007).
